# Indirect effects of a large mammalian herbivore on small mammal populations: Context‐dependent variation across habitat types, mammal species, and seasons

**DOI:** 10.1002/ece3.4670

**Published:** 2018-11-11

**Authors:** Taylor D. Ellis, J. Hall Cushman

**Affiliations:** ^1^ Department of Biology Sonoma State University Rohnert Park California; ^2^ Department of Natural Resources and Environmental Science University of Nevada Reno Nevada; ^3^ Present address: Point Reyes National Seashore California

**Keywords:** consumers, context‐dependent interactions, elk, grasslands, grazers, herbivores, indirect interactions, rodents, ungulates

## Abstract

Multiple consumer species frequently co‐occur in the same landscape and, through effects on surrounding environments, can interact in direct and indirect ways. These interactions can vary in occurrence and importance, and focusing on this variation is critical for understanding the dynamics of interactions among consumers. Large mammalian herbivores are important engineers of ecosystems worldwide, have substantial impacts on vegetation, and can indirectly affect small‐mammal populations. However, the degree to which such indirect effects vary within the same system has received minimal attention. We used a 16‐year‐old exclosure experiment, stratified across a heterogeneous landscape, to evaluate the importance of context‐dependent interactions between tule elk (*Cervus canadensis nannodes*) and small mammals (deer mice [*Peromyscus maniculatus*], meadow voles [*Microtus californicus*], and harvest mice [*Reithrodontymys megalotis*]) in a coastal grassland in California. Effects of elk on voles varied among habitats and seasons: In open grasslands, elk reduced vole numbers during fall 2013 but not summer 2014; in *Lupinus*‐dominated grasslands, elk reduced vole numbers during summer 2014 but not fall 2013; and in *Baccharis*‐dominated grasslands, elk had no effect on vole numbers in either season. Effects of elk on the two mice species also varied among habitats and seasons, but often in different ways from voles and each other. In fall 2013, elk decreased mice abundances in *Lupinus*‐dominated grasslands, but not in *Baccharis*‐dominated or open grasslands. In summer 2014, elk decreased the abundance of harvest mice consistently across habitat types. In contrast, elk increased deer‐mice numbers in open grasslands but not other habitats. Within the same heterogenous study system, the influence of elk on small mammals was strongly context‐dependent, varying among habitats, mammal species, and seasons. We hypothesize that such variability is common in nature and that failure to consider it may yield inaccurate findings and limit our understanding of interactions among co‐occurring consumers.

## 1 INTRODUCTION

Multiple consumer species commonly co‐occur in the same ecological systems and can interact with each other directly, and also in a variety of indirect ways, through their effects on surrounding plants and the community. One consumer species may impact a second by altering a shared host plant's abundance (Brown, Whitham, Ernest, & Gehring, 2001; Howe, Zorn‐Arnold, Sullivan, & Brown, 2006), distribution (Pringle, Young, Rubenstein, & McCauley, 2007), phenology (Brody, 1997; Karban & Baldwin, 1997), morphology (Huntzinger, Karban, Young, & Palmer, 2004; Nakamura, Miyamoto, & Ohgushi, 2003; Nozawa & Ohgushi, 2002; Strauss, 1991), and/or chemistry (Anderson, Sadek, & Wäckers, 2011; Denno et al., 2000; Kaplan, Sardanelli, Rehill, & Denno, 2011; Martinsen, Driebe, & Whitham, 1998; Masters & Brown, 1992). Different plant consumer species can also interact through their impacts on the plant community (Brown & Heske, 1990; Keesing, 1998; Pringle et al., 2007; Smit et al., 2001). For example, by influencing overall vegetation height, large herbivores can affect small mammals by altering their susceptibility to predators (Hagenah, Prins, & Olff, 2009; Peles & Barrett, 1996; Smit et al., 2001) or altering the availability of their food resources (Keesing, 1998).

The indirect effects of one plant consumer on another will likely vary greatly in occurrence and importance, depending on a range of temporal and spatial factors in the landscape. Numerous experiments have revealed the importance of context‐dependent variation in the outcome of indirect interactions among consumers (Maclean, Goheen, Doak, Palmer, & Young, 2011; Pringle et al., 2007). For example, differences in soil type can influence the nature of indirect interactions between consumer species by altering the susceptibility of a shared host plant to herbivory (Brown et al., 2001; Cobb et al., 1997). Biotic factors, such as the introduction or range expansion of a non‐native plant species, can also alter the indirect interactions between consumers in the invaded areas (Pearson, 2009). The consequences of indirect interactions can also depend on climatic conditions that shift over time (Brown et al., 2001; Heske, Brown, & Mistry, 1994; Long, Wambua, Goheen, Palmer, & Pringle, 2017). This variability in outcomes, combined with the rapidly changing global climate (Rosenzweig et al., 2008) and the increasing prevalence of exotic species in ecosystems (Hellmann, Byers, Bierwagen, & Dukes, 2008; Rosenstock, 1996), make long‐term experiments of critical importance for examining context‐dependent interactions among consumers. Failure to consider such variability may limit our understanding of interactions among co‐occurring consumer species (Chamberlain, Bronstein, & Rudgers, 2014).

Host‐plant‐mediated indirect interactions among consumers have been shown to arise among insects, between mammals and insects (Ohgushi, 2005; Wilkerson, Roche, & Young, 2013), and between large and small mammalian consumers (Parsons, Maron, & Martin, 2013; Smit et al., 2001). Although multiple consumers frequently co‐occur across a range of habitat types, few studies have examined if and how such environmental heterogeneity alters the outcome of their interactions. The outcome of an interaction between species may remain fairly consistent across different habitat types, or it may be strong in one habitat and weak, absent, or reversed in another. Thus, stratifying ecological experiments across multiple habitat types is critical for assessing the importance of variability in the outcome of interactions between co‐occurring consumers (Foster, Barton, & Lindenmayer, 2014).

Both large and small mammalian herbivores are important engineers of ecosystems worldwide, and they often share plant resources across a wide variety of habitats. Large mammalian herbivores are known to shape vegetative cover (Hagenah et al., 2009; Huntly, 1991; Pellegrini, Pringle, Govender, & Hedin, 2017), and small mammals can be influenced by the structure and composition of vegetation because it provides food, shelter, and protection from predators (Dutra, Barnett, Reinhardt, Marquis, & Orrock, 2011; Peles & Barrett, 1996). Positive correlations often emerge between vegetative cover and small mammal abundance (Bueno, Ruckstuhl, Arrigo, Aivaz, & Neuhaus, 2011; Hagenah et al., 2009; Keesing, 1998; Orrock, Witter, & Reichman, 2008; Pitts & Barbour, 1979; Smit et al., 2001), raising the possibility that large mammalian herbivores can affect small mammals through impacts on vegetative structure. Small mammals, in turn, can alter their host communities through seed predation (Dangremond, Pardini, & Knight, 2010; Maron & Simms, 2001), herbivory (Howe et al., 2006), and by hosting parasites that transmit disease (Keesing & Young, 2014; Keesing, Allan, Young, & Ostfeld, 2013).

In this study, we used a 16‐year‐old experiment, stratified across three habitat types, to examine the importance of and variation in indirect interactions between tule elk (*Cervus canadensis nannodes*) and three co‐occurring species of small mammals—deer mice (*Peromyscus maniculatus*), meadow voles (*Microtus californicus*), and harvest mice (*Reithrodontymys megalotis*). We addressed three questions: (a) Do elk alter the height and biomass of herbaceous vegetation that can be important to small mammal populations? (b) Do elk alter the densities of small‐mammal populations, and do these effects vary among habitat types, small mammal species or over time? (c) By altering the population density of small mammals, do elk indirectly affect seed predation rates, and do these effects vary among habitat types? Through their activities as herbivores and disturbance agents, we predict that elk will reduce the amount of vegetation and in turn reduce the abundance of small mammals. We further predict that these effects will vary substantially among habitat types, with the influence of elk on small mammals being more pronounced in open grasslands than in shrub‐dominated grasslands that provide greater protection from predators. Finally, we predict that, by reducing small‐mammal populations, elk will reduce seed predation rates for a dominant nitrogen‐fixing shrub.

## 2 METHODS

### 2.1 Study system

We performed this study on Tomales Point, a 1,030 ha peninsula that is part of Point Reyes National Seashore, 65 km northwest of San Francisco, California, USA. The vegetation of Tomales Point is a mosaic of shrub‐dominated coastal grassland and scrub, interrupted by steep canyons containing dense riparian shrubs (Lathrop and Gogan (1985). Three distinct habitat types occur within our 300‐ha study area: *Baccharis*‐dominated grasslands, *Lupinus*‐dominated grasslands, and open grasslands. Open grasslands occur on the Kehoe soil formation (derived from Cretaceous granitic parent rock; (Kashiwagi, 1985) and are dominated by herbaceous species and largely devoid of shrubs (Johnson & Cushman, 2007). *Baccharis*‐dominated grasslands occur on a subvariant of the Kehoe formation (Kashiwagi, 1985) and are characterized by herbaceous‐dominated patches mixed with dense stands of *Baccharis pilularis* (Asteraceace), a long‐lived native shrub. *Lupinus*‐dominated grasslands are located on a mix of soil formations, either completely in Sirdrak sand (derived from a Quaternary dune sandstone parent rock) or a mixture of Sirdrak sand and Kehoe variant (Kashiwagi, 1985). The latter soils are extremely well‐drained, resulting in much drier conditions in the *Lupinus*‐dominated grasslands than in *Baccharis*‐dominated or open grasslands (V. J. Dodge, V. T. Eviner & J. H. Cushman, *unpublished data*). *Lupinus*‐dominated grasslands are predominantly open areas interspersed with a short‐lived, native, nitrogen‐fixing shrub, *Lupinus arboreus* (Fabaceae), and have more bare ground than the other two habitats (J. H. Cushman, *unpublished data*). All three of these habitat types have a high proportion of overlap in herbaceous species (Johnson & Cushman, 2007).

Tule elk are native to coastal and central California (including Point Reyes) and dominated the region for centuries. However, the subspecies underwent catastrophic population declines in the 19th century due to intensive hunting and land conversion, as California experienced a large influx of Europeans during and after the Gold Rush (McCullough 1969). The dramatic decline prompted efforts to protect elk, bolster their numbers, and re‐establish populations. Tule elk have been reintroduced to numerous areas in California during the 20th century and, in 1978, eight females and two males were reintroduced to Tomales Point from a population in the San Luis Island Wildlife Refuge (Lathrop & Gogan 1985). Following a period of rapid population growth during the first two decades after reintroduction, elk at Tomales Point reached a population size of approximately 450 individuals in 1998. Since then, the herd size has fluctuated between 400 and 600 animals, although in 2014 numbers declined to below 300, possibly due to drought (D. Press, *unpublished data*). The diet of tule elk at this site consists primarily of herbaceous forbs and grasses, but they also consume shrub foliage during the winter months when there is less herbaceous vegetation available (Gogan & Barrett 1995; Johnson & Cushman, 2007).

The native small mammals that inhabit Tomales Point include the California meadow vole (*M. californicus)*, deer mouse (*P. maniculatus*), and the western harvest mouse (*R. megalotis*; Evens, 2008). California meadow voles, active both day and night, consume grasses, forbs, and soft seeds. They create runways through grassy areasand breed when grasses are fresh and sprouting (Cudworth & Koprowski, 2010). Deer mice, primarily nocturnal animals, are omnivorous, eating seeds, insects, fungi, and herbaceous plants (Jameson & Peeters, 2004). Harvest mice are also nocturnal and primarily granivorous. Typically found in grassy habitats, they often use the same runways made by voles (Webster & Jones, 1982), but their populations can negatively respond to high population densities of voles (Heske, Ostfeld, & Lidicker, 1984). The relative abundances of these rodents across the three habitats were not known prior to this study.

### 2.2 Elk exclosure experiment

This study centers around an elk exclosure experiment on Tomales Point in Point Reyes National Seashore. Established by the National Park Service and U.S. Geological Service in 1998, the experiment occurs within a 300‐ha area and consists of 24 36 × 36 m plots distributed equally among three habitat types—*Baccharis*‐dominated grassland, *Lupinus*‐dominated grassland, and open grassland. Each plot in the experiment is located 350–850 m from the Pacific Ocean. Within each of the three habitat types, there are four pairs of plots, with plots within pairs randomly assigned fencing to exclude elk or left unfenced to serve as controls. The control and exclosure plots within a pair are adjacent to one another, separated by a 3‐m wide buffer to reduce edge effects. The fencing that surrounds each exclosure plot is 2.5‐m tall and effectively excludes elk (Johnson & Cushman, 2007). Smaller herbivores at the site, such as hares (*Lepus californicus*) and the small‐mammal species mentioned previously, are able to pass easily through the fence. Black‐tailed deer (*Odocoileus hemionus columbianus*) also enter the exclosures, although probably in reduced numbers. Predators of small mammals, such as badgers (*Taxidea taxus*), coyotes (*Canis latrans*), and bobcats (*Lynx rufus*) are also able to enter the exclosures (T. D. Ellis & J. H. Cushman, *personal observation*). Other studies using this exclosure experiment have shown that elk exert major influences on ground‐dwelling arthropods (E. M. Cecil, M. J. Spasojevic, & J. H. Cushman, *unpublished data*), plant invasions (Ender, Christian, & Cushman, 2017), plant functional traits (Lee, Spasojevic & Cushman, *unpublished data*), plant community composition (Johnson & Cushman, 2007; Richter, Spasojevic & Cushman*, unpublished data;* Lee, Spasojevic & Cushman, *unpublished data*), and soil characteristics (Dodge, Eviner & Cushman, *unpublished data*).

### 2.3 Small‐mammal populations

To assess the influence of elk on small‐mammal populations, we placed 25 Sherman live traps (8 × 9 × 23 cm, H. B. Sherman Traps, Inc., Tallahassee, FL) in a 20 × 20 m grid centered within each of the 24 plots of the experiment (traps were not placed along the edges of the plots to avoid edge effects). We trapped each exclosure at the same time as its neighboring control plot. During three‐night trapping sessions, we sampled 4–6 plots per session until all 24 plots had been sampled (6–7 weeks; 1,800 trap‐nights). The first round of trapping occurred between 26 October and 14 December 2013, whereas the second round took place between 13 June and 9 August 2014. By trapping in two different seasons over a year, we were able to test whether the effect of elk on rodents varied over two time periods that differed in rainfall, length of day, food availability, life cycle stages of the elk and rodent population, and other factors.

In each plot, we pre‐baited (without setting) all traps for one night to acclimate the animals to the traps. We then trapped in each plot pair for three consecutive nights. For each captured animal, we recorded its trap location, species, and weight before releasing it at the site of capture. *Peromyscus* and *Reithrodontomys* individuals were fitted with a numbered Monel ear tag (National Band and Tag Company, Newport, KY, USA). Ear tagging allowed us to identify individuals that were caught on multiple nights, so their behavior would not influence our estimates of relative abundance. Due to low capture rates early in the study, we did not ear tag or record weights of voles. Small mammals in this study were treated according to guidelines from the American Society of Mammalogists (Sikes & Gannon, 2011), and the methods were approved by the National Park Service (permit #PORE‐2013‐SCI‐0028) and Sonoma State University IACUC.

### 2.4 Vegetation structure

To determine the effect of elk on vegetation structure, we quantified the height and biomass of accumulated dead herbaceous vegetation, which often formed a mat‐like layer of “thatch” in the plots. To measure thatch height, we divided each plot into four quarters and, between February and April 2013, measured thatch height in two shrub‐free patches closest to the center of each quarter. In September 2014, we quantified the amount of thatch biomass on the surface of each plot by collecting, drying, and weighing four 0.25 m^2^ samples per plot. The decision to take only four biomass samples per plot was drive by our desire to minimize destructive sampling and the observation that the effect of elk on biomass was consistent and great enough that additional samples were not necessary. We collected the samples from shrub‐free patches nearest to each of the four corners of a 20 × 20 m area of each plot used for small‐mammal sampling. We dried the samples at 60°C for 48 hr and weighed them immediately upon removal from the drying oven.

### 2.5 Seed predation rates

To test the hypothesis that large mammals indirectly affect rates of seed predation by small mammals, we quantified seed‐removal rates in all plots of the experiment during November of 2014. As a bioassay, we used the seeds of bush lupine (*Lupinus arboreous*), a shrub that dominates the Tomales Point study and whose seeds are regularly consumed by small mammals (Maron & Simms, 2001; Pitts & Barbour, 1979). This shrub drops most of its seeds in late summer when pods dry out and burst, and continues to drop seeds throughout the winter as the remaining seeds in the pods are dislodged by wind, rain, and other disturbances. We placed two seed depots 5 m from either side of the center of each plot in the experiment. Each depot consisted of a round plastic food storage container (12 × 20 cm) with a 2.5 cm diameter hole cut into the side to allow small mammals to enter. The hole was 2.5 cm above the bottom of the container to ensure that seeds would not easily roll out of the depot. Each container had a lid to protect the seeds from wind and disturbance by larger animals. We placed 25 lupine seeds in each container and assumed that all missing seeds had been consumed by small mammals. This assumption was supported by frequent observations of rodent feces in depots with missing seeds. After 18 days in the field, we checked the depots to quantify seed removal. We pooled the number of seeds removed from the two depots in each plot, to avoid pseudo‐replication. We occasionally found insects and isopods in the depots, but we saw no evidence that they were able to remove or consume the seeds. All seeds used in the seed predation experiment were collected within 20 km of the study site at Point Reyes National Seashore.

### 2.6 Statistical analyses

We analyzed data on small‐mammal abundances, mean weights of captured deer and harvest mice in summer 2014, seed‐removal rates, aboveground thatch biomass, and thatch height using linear mixed models, with elk (present, excluded), habitat type (*Baccharis*‐dominated*, Lupinus*‐dominated*,* open grasslands), and their interaction as fixed effects and plot pair (1–12) nested within habitat type as a random effect. For small‐mammal abundances, we also nested trap (1–25) within plot pair as a random effect, included moon phase during each trap session as a covariate and tested for an interaction between elk and moon phase. We recorded moon phase as the percent illumination according to the Astronomical Observations Department, U.S. Naval Observatory, Washington, D.C. For meadow vole abundances, we included season (fall, summer) as a fixed effect to allow for comparison across the two sampling sessions, to capture variation due to any temporal factors such as rainfall or different points in the reproductive cycle of the voles. We measured seed predation rates as the number of bush lupine seeds removed from the depots in each plot over 18 days. In these models, we initially included the following interaction terms: elk × habitat type, elk × season, elk × moon phase, elk × moon phase × habitat type, and elk × habitat type × season. If any fixed effects other than elk, habitat type, season, or the elk × habitat type interaction yielded *p*‐values exceeding 0.15, we removed them from the model, beginning with the higher‐order interactions (Crawley, 2014). We used the Kenward–Roger method (Kenward & Roger 1997) to estimate error degrees of freedom, which is widely recognized as the most rigorous approach when using linear mixed models (Kenward & Roger 1997; Schaalje, McBride & Fellingham 2002; Bolker *et al*. 2009). To ensure that assumptions for linear mixed models were met, we visually assessed all model residuals for approximate normality and checked for homoscedasticity of residual plots. If habitat type or any interaction terms were significant in our models, we followed up with Tukey multiple comparison tests to assess differences among means.

When evaluating small mammal abundance, we used the minimum numbers of mice (as determined by ear tagging over the three nights) and meadow voles (as determined by maximum number captured in one night) known to be active in each plot during each 3‐day trapping session (Slade & Blair, 2000). For 2013, we combined capture data for deer mice and harvest mice, as we did not always differentiate between these species. In 2014, we recorded harvest mice and deer mice separately and tested to determine if responses to elk differed across mouse species. Only one animal tagged in fall 2013 was recaptured in summer 2014. Since mouse species were not differentiated in 2013, we did not compare their abundances across seasons.

## 3 RESULTS

### 3.1 Small‐mammal abundance

This study involved 843 small‐mammal captures over a total of 3,600 trap‐nights. In the fall of 2013, we captured and tagged 266 mice (including deer mice and harvest mice). In summer 2014, we tagged 143 mice, of which 109 were deer mice (76%) and 34 were harvest mice (24%; Table 1). We captured meadow voles 80 times in 2013 and 136 times in 2014, with a minimum of 37 and 62 unique individuals in each year, respectively. We rarely trapped shrews (*Sorex* spp., 10 occasions) or the Point Reyes jumping mouse (*Zapus trinotatus orarius*, one occasion) and thus did not include these species in our analyses.

**Table 1:** Mean number of captured individuals of *Peromyscus maniculatus, Reithrodontymys megalotis,* and *Microtus californicus* over 75 trap‐nights per plot in summer 2014 as a function of habitat type

Mean (±1 *SE*) number of captured individuals (75 trap‐nights per plot)			
Species	*Baccharis‐*dominated grasslands	*Lupinus*‐dominated grasslands	Open grasslands
*Peromyscus maniculatus*	5.0 (±0.78)	5.63 (±1.24)	3.0 (±0.78)
*Reithrodontymys megalotis*	1.75 (±0.62)	0.5 (±0.19)	2.0 (±0.73)
*Microtus californicus*	0.88 (±0.35)	2.5 (±1.48)	3.89 (±1.32)

**Figure 1:**
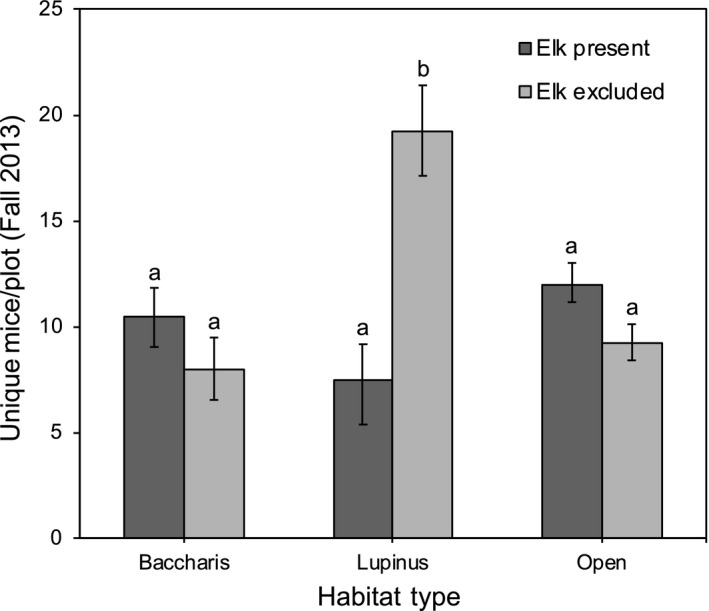
Mean maximum number of mice captured per plot per night (least known alive) in October‐December 2013, as a function of elk treatment (present, excluded) and habitat type (*Baccharis*‐dominated*, Lupinus*‐dominated*,* and open grasslands). Error bars indicate ±1 *SE*. Letters above bars correspond to the results from Tukey multiple comparison tests of least square means

In the fall of 2013, when captures of the two mouse species were pooled, we found that abundances were significantly affected by elk (*F*_1,297_ = 5.18, *p* = 0.0236) and showed a trend to vary among habitat types (*F*_2,9_ = 3.07, *p* = 0.0965). We detected a significant elk × habitat type interaction (*F*_2,297_ = 32.35, *p* < 0.0001), with multiple comparison tests indicating that elk decreased mouse abundances in *Lupinus* grasslands, but not in *Baccharis* or open grasslands (Figure 1).

In the summer of 2014, elk decreased the abundance of harvest mice (*F*_1,297_ = 8.80, *p* = 0.0033), but there was not an interaction between elk and habitat type (*F*_2,297_ = 1.79, *p* = 0.1692, Figure 2a) and abundances did not vary across habitats (*F*_2,9_ = 1.64, *p* = 0.2477).

**Figure 2:**
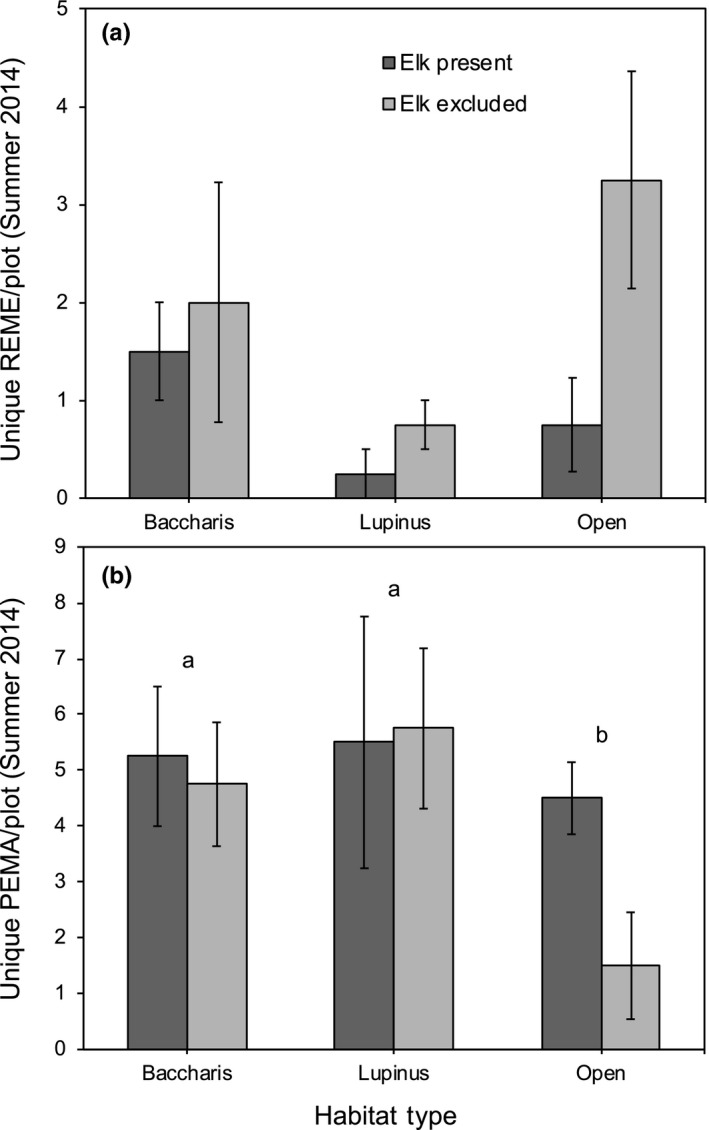
Mean numbers of individual (a) harvest mice (REME, *Reithrodontomys megalotis)* and (b) deer mice (PEMA, *Peromyscus maniculatus)* tagged in each plot from June to August 2014, as a function of elk treatment (present, excluded) and habitat type (*Baccharis*‐dominated*, Lupinus*‐dominated*,* and open grasslands). Error bars indicate ±1 *SE*. Letters above bars correspond to the results from Tukey multiple comparison tests of least square means

While elk alone did not have a strong influence on deer mouse abundance in 2014 (*F*_1,297_ = 2.56, *p* = 0.1105), there was a trend for an elk × habitat type interaction (*F*_2,297_ = 2.61, *p* = 0.0754), with elk increasing numbers in open grasslands but not other habitat types. Deer mouse abundances also varied among habitat types (*F*_2,9_ = 4.40, *p* = 0.0465; Figure 2b).

The body mass of deer mice in 2014 was not influenced by elk (*F*_1,6.9_ = 1.95, *p* = 0.2055) or habitat type (*F*_2,7.5_ = 2.79, *p* = 0.1241), but there was an interaction between the two (*F*_2,6.7_ = 6.72, *p* = 0.0225), with elk having a positive effect on the animal weight in open grasslands (Table 2). The weights of harvest mice were unaffected by elk (*F*_1,1.9_ = 4.85, *p* = 0.1683) and habitat type (*F*_2,6.9_ = 0.93, *p* = 0.4388), and there was not an elk × habitat interaction (*F*_2,1.85_ = 1.04, *p* = 0.4977; Table 2).

**Table 2:** Mean weights (g) of *Peromyscus maniculatus* and *Reithrodontymys megalotis* captured in summer 2014, as a function of elk and habitat type

Mean weights (g)				
Species	Elk treatment	*Baccharis*‐dominated grasslands (*SE*)	*Lupinus*‐ dominated grasslands (*SE*)	Open grasslands (*SE*)
*Peromyscus maniculatus*	Elk present	18.69 (±1.22)	19.35 (±1.50)	19.75 (±1.56)
	Elk excluded	20.08 (±1.67)	22.36 (±1.42)	9.56 (±1.25)
*Reithrodontymys megalotis*	Elk present	11.13 (±0.94)	12.0 (±0.0)	10.43 (±0.30)
	Elk excluded	9.48 (±1.23)	11.63 (±1.77)	10.32 (±0.96)

**Figure 3:**
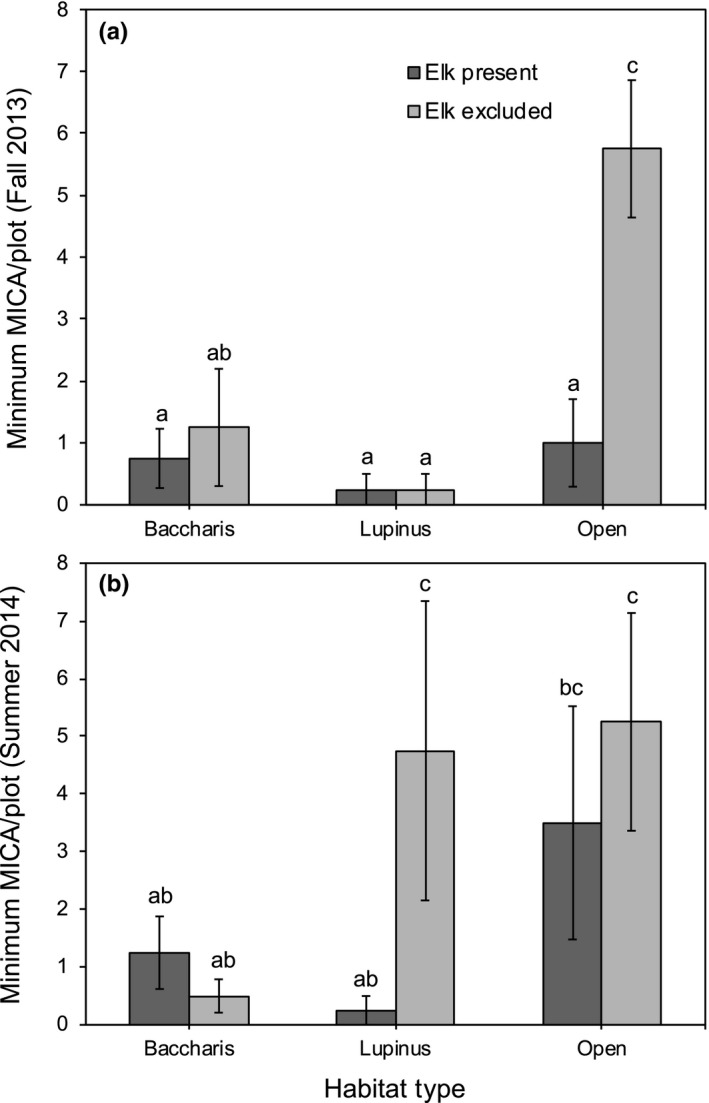
Mean maximum number of meadow voles (MICA, *Microtus californicus)* captured per plot per night (least known alive) in (a) October‐December 2013 and (b) June‐August 2014, as a function of elk treatment (present, excluded) and habitat type (*Baccharis*‐dominated*, Lupinus*‐dominated*,* and open grasslands). Error bars indicate ±1 *SE*. Letters above bars correspond to the results from Tukey multiple comparison tests of least square means from both seasons

As shown in Figure 3, the abundance of meadow voles was reduced by elk (*F*_1,892_ = 31.56, *p* = 0.0011) and varied across habitat types (*F*_2,9_ = 8.37, *p* = 0.0088). In addition, we detected significant elk × habitat type (*F*_2,892_ = 9.85, *p* < 0.0001) and elk × habitat type × season interactions (*F*_2,892_ = 12.62, *p* < 0.0001; Figure 3). Multiple comparison tests showed that elk reduced vole numbers in open grasslands during fall 2013 but not summer 2014, and in *Lupinus*‐dominated grasslands during summer 2014 but not fall 2013. Voles in *Baccharis*‐dominated grasslands were unaffected by elk in either year.

### 3.2 Seed removal

**Figure 4:**
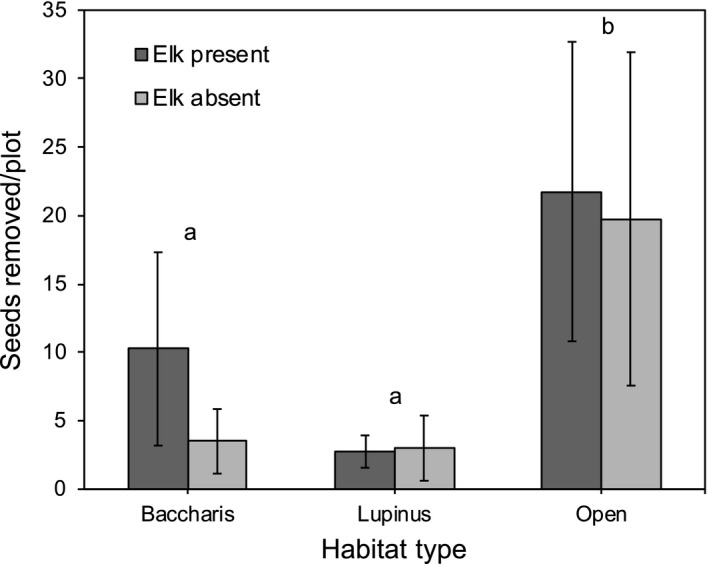
Mean number of *Lupinus arboreus* seeds removed per plot in November 2014, as a function of elk treatment (present, excluded) and habitat type (*Baccharis*‐dominated*, Lupinus*‐dominated*,* and open grasslands). Error bars indicate ±1 *SE*

Contrary to our prediction, elk did not influence the removal of *L. arboreus* seeds (*F*_1,7.3_ = 0.73, *p* = 0.4193), and there was not an elk × habitat type interaction (*F*_2,7.3_ = 0.42, *p* = 0.6707; Figure 4). Seed removal did vary among habitat types (*F*_2,4_ = 21.90, *p* = 0.0071), with significantly more seeds being removed in open grasslands than in either *Baccharis*‐dominated or *Lupinus*‐dominated grasslands (Figure 4).

### 3.3 Vegetation structure

**Figure 5:**
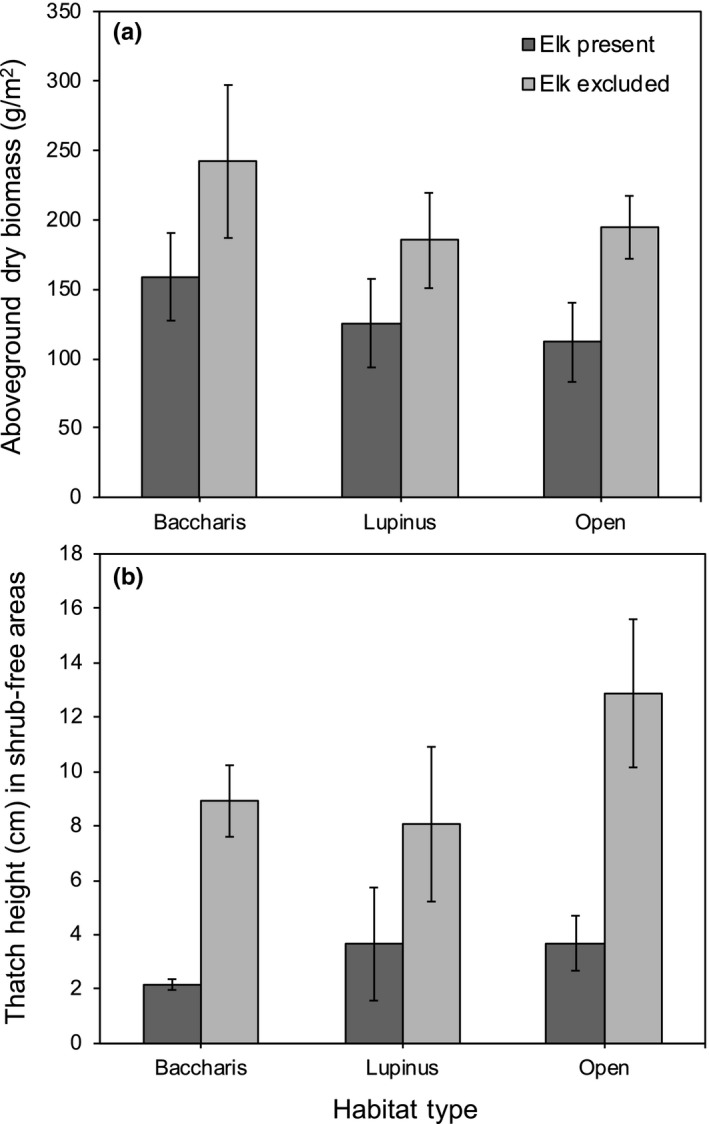
Mean (a) aboveground dry herbaceous biomass and (b) thatch height as a function of elk treatment (present, excluded) and habitat type (*Baccharis*‐dominated*, Lupinus*‐dominated*,* and open grasslands). Error bars indicate ±1 *SE*. Letters above bars correspond to the results from Tukey multiple comparison tests of least square means

Elk significantly reduced thatch biomass (*F*_1,9_ = 8.88, *p* = 0.0155), but amounts did not vary among habitat types (*F*_2,9_ = 0.91, *p* = 0.4358) nor was there an elk × habitat type interaction (*F*_2,9_ = 0.10, *p* = 0.9093, Figure 5a). Elk also reduced the height of thatch in shrub‐free areas (*F*_1,9_ = 19.92, *p* = 0.0016), although thatch height did not vary among habitat types (*F*_2,9_ = 1.66, *p* = 0.2432) and the elk × habitat type interaction was insignificant (*F*_2,9_ = 0.25, *p* = 0.7831, Figure 5b).

## 4 DISCUSSION

Multiple consumer species frequently co‐occur across a range of habitat types, and it is important to understand if and how their interactions change due to this environmental heterogeneity. Using a 16‐year‐old exclosure experiment stratified across three habitat types, we have shown that the outcome of interactions between co‐occurring mammal species is highly context‐dependent. Although tule elk reduced the height and biomass of herbaceous vegetation in all three habitat types, their effects on small‐mammal abundances commonly varied among habitat types, rodent species, and season. Elk reduced numbers of harvest mice, but not deer mice, in 2014, although deer mice showed a trend to be affected by elk in open grasslands. Meadow voles showed the greatest negative response to elk, but were unaffected in *Baccharis*‐dominated grasslands and only seasonally affected in *Lupinus*‐dominated and open grasslands.

The effects of elk on small‐mammal populations varied among species, which could reflect the relative importance of herbaceous cover to the different species (Bakker, Olff, & Gleichman, 2009). Tule elk reduced aboveground herbaceous biomass and thatch height in all habitat types, but did not always suppress rodent populations. Studies by Heske and Campbell (1991), Keesing (1998), and Pedersen et al. (2014) also failed to find a link between rodent abundance and vegetative cover, possibly because factors other than refuge from predation were more important drivers of small‐mammal abundance in those systems. While elk reduced meadow vole abundance in open grasslands, they increased deer mice numbers in this habitat. A similar outcome was documented by Bueno et al. (2011), who found that vole numbers sharply declined in areas grazed by cattle, while those for deer mice increased. We considered the possibility that interactions between voles and mice might be affecting their abundances, but found no evidence of this in our system. Meadow voles (Cudworth & Koprowski, 2010) and harvest mice (Webster & Jones, 1982) are especially associated with grassy habitats, but deer mice occur in a wider range of habitats (Jameson & Peeters, 2004) and may be less dependent on the protection from predators offered by herbaceous plants. Food availability can be a more important driver of rodent numbers than the amount of protective vegetation (Keesing & Young, 2014), and elk may sometimes facilitate growth of food plants preferred by deer mice, despite reducing overall biomass of herbaceous vegetation (Arsenault & Owen‐Smith, 2002).

Vole populations can rapidly increase when there is an abundance of fresh vegetation (Cudworth & Koprowski, 2010), and this may explain why their numbers were greater in the summer of 2014 than the fall of 2013 (Figure 3). Winter rains prompt new plant growth in this grassland, and our fall sample was taken just prior to the rainy season, when conditions were exceptionally dry. Both sampling periods occurred during a persistent drought in California, which probably increased competition among rodents for limited food resources. We also found that voles were only seasonally affected by elk in *Lupinus*‐dominated and open grasslands. In 2013, there were few voles captured in *Lupinus*‐dominated grasslands (Figure 3a), but in 2014, their numbers increased greatly in the *Lupinus* exclosures, while remaining low in *Lupinus* controls (Figure 3b). Vole numbers in open grasslands were robust in both seasons, but in summer 2014, we found no effect of elk on their abundance there (Figure 3b). This lack of an effect could be due to substantial plot‐level variation in vole abundance in open grasslands during the summer of 2014, which made the effects of elk difficult to detect with our sample size (*n* = 8 plots per habitat). Our study suggests that elk inhibit voles from experiencing the population spikes that would otherwise occur in an ungrazed system, but that high‐density populations of voles are still able to arise in ungrazed, grassy habitats when conditions support such population increase.

Our study complements other exclosure experiments that have examined the impacts of large herbivores on small‐mammal populations. Large mammalian herbivores have been shown generally to reduce the abundance of small mammals that compete with them for food sources, although this effect diminishes in more productive ecosystems (see Daskin and Pringle, 2016 for a review). Of particular relevance to our work is a study by Parsons et al. (2013), who found that elk in a forested habitat reduced the abundance of voles more than deer mice. Although their experiment was not stratified across different habitat types, they found that small mammals responded to microhabitat features, such as shrub cover, that were altered by elk. Another herbivore‐exclusion experiment by Smit et al. (2001) found that rodent numbers varied among different forest habitats and were reduced by red deer (*Cervus elaphus*) roe deer (*Capreolus capreolus*), and wild sheep (*Ovis ammon musimin*) in the Netherlands. Although they did not statistically test for an interaction between large herbivores and habitat type, they found that rodents responded more quickly to herbivore exclusion than to herbivore introduction, presumably because vegetation recovered quickly when herbivores were excluded but took more time to become degraded when herbivores were introduced. Pedersen et al. (2014), testing for an interaction of large herbivores with wildfire on small mammal abundance in Australia, found that deer reduced the abundance of two small mammal species, but only in recently burned areas and not in older burned areas. Young et al. (2015) found context‐dependent effects of native large herbivores on small mammal populations in Kenya. Small mammals in this study tended to increase in abundance after the removal of large herbivores, but the effect was smaller in areas that were used for agriculture and there was no effect in areas with livestock grazing. The authors also found a significant rainfall × land‐use interaction, with rainfall affecting the responses of small mammal in agricultural and pastoral areas but not in areas without agriculture or livestock. Long et al. (2017) detected an interaction between large herbivores and rainfall on habitat selection by small mammals in Kenya. That study pooled captures of three species of small mammals, from both bare ground and tree‐covered patches, in plots that were either accessible to large herbivores or from which large herbivores had been excluded. Collectively, small mammals in that study were more likely to avoid open patches of ground and favor areas of tree cover in plots with large herbivores than in exclusion plots, where they showed less preference between patches. While this effect occurred during both wet and dry seasons, it was stronger during the dry season. The variation in these results illustrates the importance of considering the context of consumer interactions and points to potential limitations in the predictive ability of unstratified experiments. Here, we tested for an elk × habitat interaction and showed that habitat characteristics can significantly alter the effect of elk on small mammal abundance.

Small mammals can alter their host communities through seed predation (Dangremond et al., 2010; Maron & Simms, 2001), raising the possibility that large mammals might indirectly affect plant communities through their effects on the abundance of granivorous small mammals. However, we did not detect an effect of elk on seed predation rates, even though they commonly altered rodent abundances. Our results contrasted with those of Smit et al. (2001) who found that elk, deer, and wild sheep reduced seed predation rates, presumably because they also reduced the abundance of wood mice (*Apodemus sylvaticus*) and field voles (*Microtus agrestis*). Seed predation from our depots was often “all‐or‐nothing,” with some depots emptied quickly and others apparently unvisited for weeks. Seed‐removal rates may vary with vegetative cover (Dutra et al., 2011; Mattos, Orrock, & Watling, 2013) and among seed species (Orrock & Damschen, 2005). It is likely that local conditions surrounding each depot, such as shrub cover or the availability of alternate food sources (including *Lupinus* seeds from nearby plants), were more important determinants of seed‐removal rates than rodent abundance over the entire plot and may explain the greater seed‐removal rates observed in open grassland habitat.

Our 16‐year‐old exclosure experiment has shown that the reintroduction of a large mammalian herbivore has had substantial indirect effects on small‐mammal populations. By stratifying the experiment across three habitat types and two seasons, we have shown that elk have strongly context‐dependent effects on two of the three small‐mammal species. Although numerous studies have evaluated the interactions between co‐occurring consumers, fewer have examined their indirect interactions, and fewer still have examined the context dependency of these interactions across different habitat types, seasons, and mammal species. We hypothesize that habitat‐specific variability in consumer interactions is common in nature and that failure to focus on it may limit our understanding of interactions among co‐occurring consumer species.

## CONFLICT OF INTEREST

None declared.

## AUTHOR CONTRIBUTIONS

TDE and JHC conceived and designed the study, performed the experiment, analyzed the data, and wrote the manuscript.

## DATA ACCESSIBILITY

Data associated with this manuscript are available on Dryad at doi:10.5061/dryad.1gv57s0.
